# Estrogens in Hepatocellular Carcinoma: Friends or Foes?

**DOI:** 10.3390/cancers13092085

**Published:** 2021-04-26

**Authors:** Giuseppe Carruba

**Affiliations:** Servizio di Internazionalizzazione e Ricerca Sanitaria (SIRS), Azienda di Rilievo Nazionale e di Alta Specializzazione (ARNAS)-Civico, Di Cristina, Benfratelli—Palermo, Piazza N. Leotta 2, 90127 Palermo, Italy; giuseppe.carruba@arnascivico.it

**Keywords:** liver, estrogen, aromatase, estrogen receptors, amphiregulin, ADAM17, merlin, FOXA

## Abstract

**Simple Summary:**

Today, we know that estrogen hormones are required for the development and function of many organs, such as the liver, in both males and females. However, in some circumstances, estrogen excess may be implicated in the appearance of various chronic diseases, including cancer. This review will inspect the results of several studies to better understand the mechanisms responsible for estrogens to change from protective into harmful hormones in human liver.

**Abstract:**

Estrogens are recognized as key players in physiological regulation of various, classical and non-classical, target organs, and tissues, including liver development, homeostasis, and function. On the other hand, multiple, though dispersed, experimental evidence is highly suggestive for the implication of estrogen in development and progression of hepatocellular carcinoma. In this paper, data from our own studies and the current literature are reviewed to help understanding this apparent discrepancy.

## 1. Introduction

Estrogens play a key role in the physiology of an amazing array of systems, organs, and tissues, including reproductive, cardiovascular, central nervous systems, as well as adipose tissue, skeletal muscle, and liver, reviewed in [1]. They have been implicated in the control of food intake, energy expenditure and balance, glucose homeostasis, and insulin sensitivity, as well as in the prevention of fat accumulation and tissue inflammation.

There is dated evidence that sex hormones, notably estrogens, are important regulators of both morphology and function of human liver and that they could also be implicated in the development of various liver diseases, including cancer [2,3].

This work reviews data from literature and our own studies that may help understanding the seeming discrepancy between evidence supporting the protective role of estrogen against chronic liver diseases, on one hand, and evidence implicating estrogen in development and/or progression of hepatocellular carcinoma (HCC), on the other. In particular, studies on estrogen formation, signaling mechanisms, and stem/progenitor cell regulation in either healthy or diseased liver have been surveyed. A hypothetical model of estrogen role in both the maintenance of liver homeostasis (*friend*) and the onset of hepatic malignancies (*foe*) is herein proposed and discussed, also in the light of its potential implications not only for future therapeutic options in the management of HCC patients, but also for a better understanding of the pathogenetic mechanisms underlying an array of chronic ailments, including hormone-related cancers, endometriosis, polycystic ovary syndrome, and neurodegenerative diseases.

## 2. Estrogen Protection of Liver from Various Diseases, Including Cancer

Nowadays, it is widely recognized that the liver is sexually dimorphic, with premenopausal females having a lower risk of developing hepatic diseases and liver-driven metabolic disorders than postmenopausal women and male counterparts; furthermore, key estrogen effects on sexual-specific and metabolic functions of the liver appear to be mediated through the activation of specific hepatic estrogen receptors (ER), notably the ERα, as reviewed in [4,5].

Estrogen regulates liver metabolism and energy balance by acting through estrogen receptors [6]. In particular, estradiol decreases lipogenesis and fatty acid uptake, while it enhances lipolysis and cholesterol secretion, thus preventing lipid accumulation and liver steatosis. Furthermore, estrogens promote glucose storage in the liver by decreasing glucose production through the reduction of gluconeogenesis and the increase of glycogen synthesis, eventually leading to an improvement of glucose tolerance and insulin sensitivity [7].

The recognition that estrogens regulate liver lipid metabolism and homeostasis is also corroborated by the evidence that estrogen deficiency, as it occurs with antiestrogen treatment, after menopause or in experimental animal models, ultimately results in liver fat accumulation and steatosis.

An increased risk of developing steatohepatitis is a well-documented side-effect of the use of tamoxifen, a weak synthetic estrogen having antiestrogenic activity, in the treatment of hormone-responsive breast cancer [8]. Furthermore, a significant accumulation of lipids occurs in the liver of aromatase knockout (ArKO) mouse, with a correspondingly increased expression of lipogenic genes, including FASN and SCD-1 [9]. Interestingly, the fatty liver observed in the ArKO male mice as a consequence of estrogen deficiency could be reversed through the administration of exogenous natural estrogens and/or a specific ERα-agonist [10,11].

Using a mouse model with deletion of hepatic ERα, Palmisano and colleagues [12] observed that estrogens are no longer able to constrain liver steatosis, suggesting that they decrease the triglyceride liver content by acting directly through ERα. In addition, consistent evidence indicates that estrogens suppress *de novo* hepatic lipogenesis through ERα-mediated binding to genes involved in both lipid biosynthesis and fatty acid metabolism and via a sustained phosphorylation of acetyl-CoA carboxylase (ACC) [13,14].

Estrogens also decrease cholesterol biosynthesis and negatively regulate its uptake in the liver through an ERα-mediated mechanism [15]. In addition, estrogens stimulate the biosynthesis of both lipoproteins and proteins responsible for blood coagulation (such as factors II, VII, IX, X, and plasminogen) in the liver [16].

It is worth noting that the important protective effects of estrogens on liver lipid metabolism could represent an indirect consequence of estrogen-induced restriction of fatty acid (FA) release from adipose tissue and, hence, the resulting decrease of FA hepatic delivery [17].

There is convincing evidence that estrogens play a protective role following traumatic injury and the resulting shock/sepsis in a variety of systems and organs [18]. As far as the liver is concerned, it has been observed that estrogen downregulates the production of proinflammatory cytokines by Kupffer cells in response to trauma-hemorrhage [19]. The protective effect of estradiol on liver injury after trauma-hemorrhagic shock has also been ascribed to a p38 mitogen activated protein kinase (MAPK)-dependent up-regulation of hepatic hemeoxygenase-1 (HO-1) expression [20]. In addition, estradiol administration after trauma-hemorrhage induces expression of heat shock protein (HSP) 32 and 70 in the injured liver through an ERα-mediated mechanism, suggesting that this beneficial effect of estrogen on hepatic function could at least partly be mediated by HSP induction [21].

In a recent paper, Charni-Natan and associates [22] have proposed that p53, the renowned tumor suppressor gene, has an important role in the regulation of steroid hormones, including estrogens, and in the preservation of liver homeostasis, suggesting that p53 may function as a key intermediary and a novel regulator in this axis.

In the last decades, multiple, though sparse, evidence has accumulated suggesting that estrogens are key players in liver protection from various diseases, including fibrosis, non-alcoholic fatty liver disease (NAFLD), chronic hepatitis, and hepatocellular carcinoma (HCC).

In a retrospective study of patients with biopsy-proven chronic hepatitis C virus (HCV) disease, Villa et al. [23] observed that progression of liver fibrosis in post-menopausal women is directly related to the extent of estrogen deprivation and the reduction of estradiol/testosterone ratio. Furthermore, the expression of ERα has been reported to be inversely related to liver fibrotic stage in patients with HCV genotype 1b infection [24].

A study by Yang and coworkers indicated that men and postmenopausal women with nonalcoholic steatohepatitis (NASH) are at a higher risk for developing severe fibrosis as compared to women before menopause [25]. This effect could also be ascribed to the antioxidant activity of endogenous estradiol that overcomes hepatic fibrosis in animal models and counteracts the production of reactive oxygen species in primary cultures of hepatic stellate cells [26,27].

Consistent evidence indicates that prevalence and severity of NAFLD are greater in men than in women in the reproductive age, while, after menopause, NAFLD occurs at a higher rate in women, suggesting that estrogens play a protective role in both the development and progression of the disease, reviewed in [28,29]. Klair and colleagues [30] have suggested that postmenopausal women with NAFLD and lengthy estrogen deficiency may have a higher risk of developing liver fibrosis than premenopausal women. Postmenopausal patients with early breast cancer treated with nonsteroidal aromatase inhibitors have an increased risk of developing NAFLD as a consequence of the inhibition of estrogen biosynthesis, with a negative impact on clinical outcomes [31]. Recently, Della Torre [32] has emphasized the concept that estrogens, along the evolutionary path, have provided the female liver with high metabolic dynamicity and flexibility, eventually leading to antagonize metabolic and inflammatory alterations underlying NAFLD development and, hence, determining its lower prevalence in fertile females and increasingly high incidence associated with menopause.

Several studies have reported that both chronic hepatitis B virus (HBV) and HCV occur more frequently and progress more rapidly in males than in females and that liver cirrhosis is largely prevalent in men and women after menopause [33]. HBV infection appears to be less aggressive and to progress slower in fertile females than in males, while both NAFLD and cirrhosis occur less frequently in premenopausal women, suggesting a favorable role of estrogens against the progression of hepatic fibrosis and chronic liver diseases [33,34]. Recently, Ruggieri and colleagues [35] have suggested that sex hormones, viruses and immune response are strictly interrelated to determine the sexual dimorphism in the development and clinical outcome of both B virus and C virus chronic hepatitis diseases.

In general, estrogens promote a variety of protective mechanisms, including preservation of mitochondrial structure and function, the inhibition of cellular senescence, and the stimulation of innate immunity [36]. The protecting role reported for estradiol (E2) in chronic liver diseases, including HCC, has also been attributed to the anti-proliferative and anti-inflammatory activities brought about by E2 through binding to and activation of estrogen receptor beta (ERβ) [37]. On the other hand, in peri- or post-menopausal women, ovarian failure and the resulting estrogen deficiency may eventually lead to higher risk of developing various liver diseases, especially NAFLD and hepatocellular carcinoma, as well as faster progression of fibrosis in HCV infection [36].

The major evidence reported in this section is summarized in Table 1.

## 3. Estrogen Formation and Activity in HCC

Liver cancer represents the fifth commonest cancer in men and the ninth in women worldwide, with Eastern and South-Eastern Asia, Micronesia, West and Central Africa, and Egypt having the highest incidence rates [38]. Globally, liver cancer is the second-leading cause of cancer death in men and the sixth in women. Nearly 80% of primary liver cancers are represented by HCC. In Italy, 13,000 new HCC cases were expected in 2020, while mortality rates have been steadily decreasing since the late 1990s, with an annual decrease rate of 1.6% in males and 1.3% in females [39].

Independent of race and geography, HCC incidence is up to three times greater in male than in female, suggesting that gonadal steroids could be implicated in its development. Previous reports have suggested that sex steroids play a role in the development and progression of HCC as consequence of an unbalanced liver hormonal milieu [40]. However, studies conducted either in vitro, or in experimental animal models and HCC patients have often provided conflicting or uncertain results [41].

Early studies have reported the expression of estrogen receptors in primary HCC [40,42], suggesting a potential role of estrogens in HCC development. However, various clinical trials have failed to demonstrate any beneficial effect of the treatment with the antiestrogen tamoxifen on the clinical outcome of HCC patients [43,44,45]. More recent ER studies have reported that the ESR1 gene, which encodes for ERα, is frequently hypermethylated and that, therefore, the expression levels of ERα drop to less than 10% in HCC [46]. On the other hand, the expression of mutant ERα has been observed in primary HCC, suggesting that although variant ERs would still be transcriptionally active, they could be refractory to antiestrogen binding and, consequently, HCC would become unresponsive to tamoxifen treatment [47].

The biotransformation, conjugation, and degradation of sex steroids occur primarily in the liver through the activity of key steroid enzymes, including aromatase. There is evidence that aromatization of androgen into estrogen in human liver is associated with a higher risk of developing non-viral hepatitis-related HCC [48]. More recently, Murakami et al. [49] reported that high immunoreactivity scores of aromatase are correlated with HBV infection, suggesting a potential role for in situ estrogen biosynthesis in liver chronic B-virus hepatitis.

**Figure 1:**
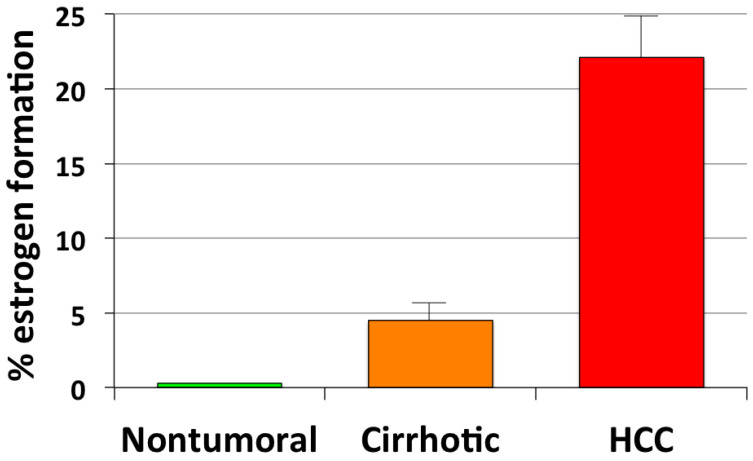
Aromatase-driven estrogen formation in human liver tissues. Aromatase activity was measured through incubation for 72 h of minced nontumoral, cirrhotic and cancer liver tissues with physiological amounts of tritiated testosterone used as androgen precursor. Values represent average percent conversion rates ±SD from triplicate experiments [50,51].

**Figure 2:**
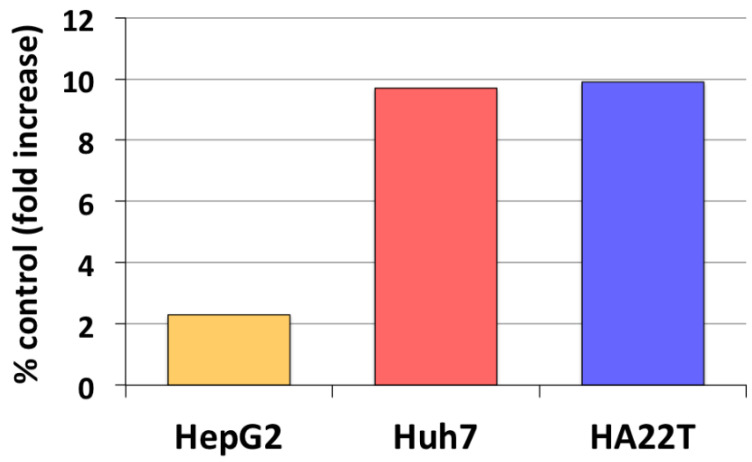
Estrogen regulation of amphiregulin (AREG) expression in liver cancer cells. Changes of AREG expression following exposure for 24 h to physiological (1 nM) estradiol were measured in HepG2, Huh7 and HA22T liver cancer cells through RT-PCR semiquantitative analysis. Results represent average percent fold increase with respect to control cell cultures receiving vehicle alone [52].

**Figure 3:**
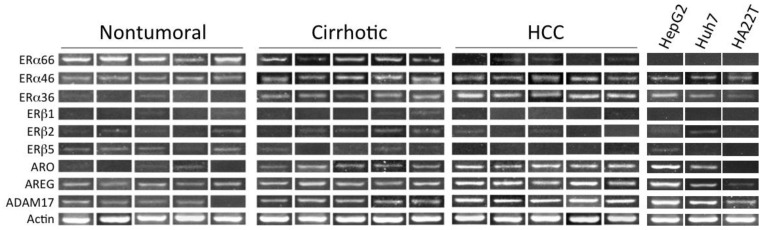
Expression of estrogen receptors, aromatase, amphiregulin, and ADAM17/TACE in liver tissues and cancer cells. Expression of estrogen receptor α (ERα) and β (ERβ), either wild-type (ERα66; Erβ1) or splice variants (ERα46, ERα36; ERβ2, ERβ5), was measured using exon-specific RT-PCR. Expression of aromatase (ARO), amphiregulin (AREG), and the disintegrin and metalloprotease 17 (ADAM17) was measured using conventional semiquantitative RT-PCR analysis. For methodological details see quoted references [54,55].

**Figure 4:**
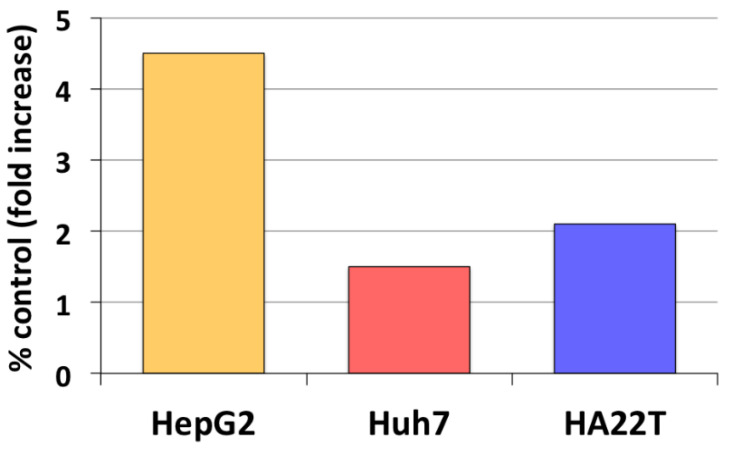
Estrogen regulation of the disintegrin and metalloprotease 17 (ADAM17) expression in liver cancer cells. Changes of ADAM17 expression following exposure for 24 h to physiological (1 nM) estradiol were measured in HepG2, Huh7 and HA22T liver cancer cells through RT-PCR semiquantitative analysis. Results represent average percent fold increase with respect to control cell cultures receiving vehicle alone [52].

**Figure 5:**
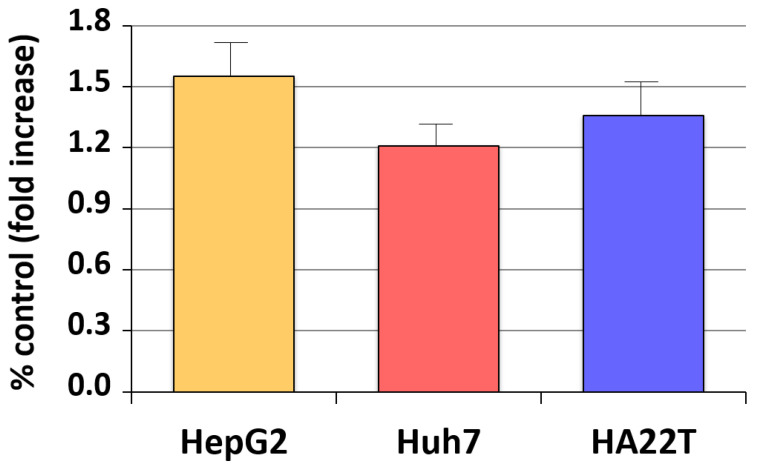
Growth regulation of liver cancer cells by amphiregulin. The figure illustrates the impact of AREG (50 ng/mL) on proliferative activity of HepG2, Huh7 and HA22T liver cancer cells. Values represent average fold increase ±SD of quadruplicate wells from triplicate experiments with respect to control cell cultures receiving vehicle alone [54].

Our earlier studies have revealed that aromatase expression and activity is higher in malignant, but markedly lower or undetectable respectively in cirrhotic and *nontumoral* tissues and cells (see Figure 1) [50,51]. We have also observed that locally elevated estrogen production results in the increase of both the expression and activity of a membrane glycoprotein, amphiregulin (AREG), that may in turn lead to stimulation of epidermal growth factor receptor (EGFR) signaling and cell proliferation (Figure 2) [52]. It is well known that AREG is activated through a process, referred to as ectodomain shedding, whereby a transmembrane metalloprotease, a disintegrin and metalloprotease 17 (ADAM17 or TACE), is responsible for AREG cleavage into distinct EGF-like fragments [53]. We have reported that both AREG and ADAM17/TACE are expressed correspondingly with aromatase in liver tissues and liver cancer cell lines (see Figure 3). Furthermore, ADAM17/TACE is induced by treatment with estradiol (E2) of liver cancer cells (Figure 4), indicating that estrogen may increase AREG activity in the malignant liver either directly (through the upregulation of the AREG gene, increase of its transcriptional activity or protein biosynthesis) or indirectly (via the induction of ADAM17/TACE enzyme) or both [52]. In this context, liver cancer cell growth is stimulated by exposure to AREG (Figure 5), while this effect is abrogated by the simultaneous addition of a neutralizing anti-AREG antibody, implying that the estrogen-induced rise of AREG expression and/or activity may ultimately lead to a significant increase of cell proliferation [54].

## 4. Estrogen and the Product of Neurofibromatosis Type 2 (NF2) Gene, Merlin

In previous studies, we have reported that, comparing nontumoral, cirrhotic and malignant liver tissues, an increasingly higher aromatase expression and activity is strictly associated with a progressive reduction of the wild type ERα (Erα66) and the gradual emergence of the ERα36 splicing variant, suggesting that this ER shift could be distinctively implicated in HCC development and progression [54,55]. In this framework, an interesting piece of evidence comes from studies on the expression and role of Merlin (neurofibromin 2), the product of the neurofibromatosis type 2 (NF2) tumor suppressor gene, in human liver. Merlin is a cytoskeletal protein belonging to the Band 4.1 family of linker proteins, containing the ERM (Ezrin, Radixin, Moesin) domain, that presides over the stabilization of the membrane-cytoskeleton interface and restricts contact-dependent EGFR mobility and internalization [56,57]. NF2 gene is a key regulator of the Hippo cellular pathway, which in turn controls organ size and various cell functions, including proliferation, motility, survival, and signaling [58]. Merlin/ERM proteins have been implicated in the control of cell signaling and growth by regulating the distribution of membrane receptors and their interaction with the underlying cytoskeleton [56].

Evidence has accumulated that although most genes belonging to the Hippo pathway are not frequently mutated, the Hippo pathway activity has been reported to be deregulated in different human cancers [59]. In this context, Merlin functions as a tumor suppressor also by impacting upon Hippo-mediated mechanisms controlling cell proliferation, apoptosis, survival, motility, adhesion, and invasion [60,61]. In particular, Merlin restricts tumor growth and reverts malignant phenotype in vitro [62,63,64,65].

**Figure 6:**
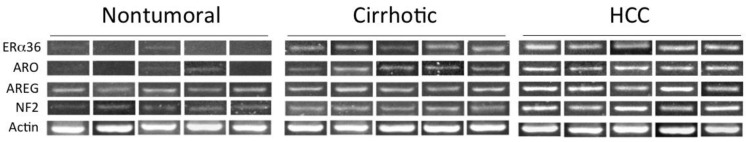
Corresponding expression of ERα36, aromatase, amphiregulin, and Merlin (NF2) in liver tissues. The expression of the ERα36 splice variant, aromatase (ARO), amphiregulin (AREG), and the product of neurofibromatosis tumor suppressor gene, Merlin (NF2), were determined respectively using exon-specific RT-PCR and conventional semiquantitative RT-PCR analysis. For methodological details see quoted reference [54].

Using conditional knockout mice carrying liver-specific deletion of NF2, Benhamouche and colleagues [66] observed a significant, progressive expansion of putative liver stem/progenitor cells, while differentiated hepatocytes were not affected. Interestingly, surviving mice invariably developed cholangiocellular (CC) and HCC, suggesting that NF2−/− oval cells could represent the tumor-initiating cells in liver carcinogenesis. In this experimental model the authors suggested that Merlin may act through a Hippo-independent stimulation of EGFR abundance and signaling, with pharmacologic inhibition of EGFR eventually leading to constrain proliferative activity of NF2−/− stem/progenitor cells both in vitro and in vivo. In our liver studies, Merlin expression is strictly associated with that of both aromatase and AREG, resulting prominent in HCC, intermediary in cirrhotic tissues and markedly lower in nontumoral liver; interestingly, NF2 is also proportionally expressed with the truncated hERα36 splice variant and inversely related to wild type hERα66 (see Figure 6) [54]. This evidence suggests that Merlin could be regulated in association with estrogen formation and signaling in both normal and diseased liver.

## 5. Estrogen Receptors in Human HCC

The presence of estrogen receptors in human liver was firstly reported by Duffy in the late 1970s [67]. From subsequent studies, multiple, though spare, evidence has indicated that estrogen receptors are variably expressed in a considerable proportion of human HCC [42,68]. This evidence has suggested that estrogens may be implicated in human liver malignancies, though their potential role in the development and/or progression of HCC has remained undefined. In this framework, the antiestrogen tamoxifen has been introduced as a potential therapeutic option for unresectable HCC [69,70,71]. However, results from several clinical trials and systematic reviews or relevant meta-analyses have shown that tamoxifen is largely ineffective in increasing overall or disease-free survival [72,73,74,75]. In this context, it is worth noting that in most clinical trials the ER status of primary HCC has been assessed using biochemical ligand binding assay and/or immunocytochemical analysis, in both cases with no indication on the expression of variant ERs. In our studies, we have observed that HCC development and progression appear to be associated with a prominent switch of the expression of ERα66 and ERα36, with the former (wild-type ER) being predominant in nontumoral liver tissues and the latter (variant ER) largely prevalent in HCC tissues [54,55]. As stated above, the ER status of primary HCC has been determined using either biochemical or immunochemical assay, also using mono- and/or polyclonal antibodies that are often raised against epitopes that are lost in the 36 kDa splicing variant of ERα. This would imply that immunocytochemically ER-negative HCC could yet express ERα36, indicating that routine evaluation of ER status based solely on immunohistochemistry could often be inadequate for a comprehensive assessment of ER status and estrogen signaling in liver tissues and cells and, therefore, could fail to provide potentially important information on ‘‘ER-negative’’ HCC patients.

As reported above, we have observed that aromatase is expressed in nontumoral, cirrhotic, and HCC liver in association with the ERα36 splice variant, while it appears to be inversely related to the wild type Erα66. The ERα36 lacks both ligand-independent (AF1) and -dependent (AF2) transactivation regions of ER but retains DNA- (DBD) ligand-binding (HBD) domains and is featured by a unique 27-amino-acid domain sequence at the COOH terminus in place of the last 138 residues encoded by exon 7 and 8 of the wild-type hERα66 gene [76]. Interestingly, the ERα36 isoform contains three potential myristoylation sites located near the N-terminus that are thought to mediate a posttranslational modification responsible for membrane localization of the receptor [77]. Although the ERα36 variant lacks intrinsic transcriptional activity, it is able to repress transactivation of wild-type ERα66, eventually leading to the inhibition of classical genomic estrogen signaling. In addition, once located on the plasma membrane, the ERα36 isoform may trigger nongenomic signaling, including the activation of MAPK/ERK pathway and the resulting increase of cell proliferation [77]. The overexpression of ERα36 has been reported in breast cancer patients having poorer disease-free survival, featuring a subset of patients who are unlikely to benefit from tamoxifen treatment [78]. In a wider context, the balance of wild-type ERα66 and the ERα36 isoform may be decisive to drive target tissues towards either growth control or deranged cell proliferation, eventually leading to the development/progression of breast cancer and other hormone-related tumors, including HCC.

Armed with this combined evidence, emerging from our own and other studies, it is plausible to hypothesize that, in human liver, locally high, aromatase-driven estrogen formation may activate ERα36-mediated nongenomic signaling that could in turn lead to sustained cell growth and, therefore, to HCC development. In particular, the prevalence of the ERα36 splice variant could result in the suppression of both hormone-dependent and -independent, ERα66-mediated, estrogen genomic effects, with the concurrent activation of rapid estrogen signaling through the MAPK/ERK pathway, as it occurs in human breast cancer [77]. Interestingly, the expression of the ERα36 isoform has been implicated in the progression of various human cancers, including renal cell carcinoma, papillary thyroid carcinoma, laryngeal carcinoma, endometrial carcinoma, and gastric cancer, reviewed in [79]. Specifically, ERα36 has been reported to up-regulate EGFR expression and down-regulate of ERα66 expression in human breast cancer cells, suggesting that this could represent an underlying mechanism for the generation of acquired tamoxifen-resistance in breast cancer patients [80].

In this context, the failure of the antiestrogen tamoxifen to increase the overall survival of HCC patients could represent, at least partly, the consequence of the aforementioned switch of the ERα66 into ERα36 expression that may occur during liver cancer development and progression. As stated above, this ERα variant has a unique 27-amino acid sequence at the COOH terminus that may result in an alteration of the ligand binding domain and, therefore, of binding affinity and specificity of the receptor. Specifically, Wang and colleagues [77] reported that both tamoxifen and the “pure” antiestrogen ICI-182,780 fail to block the ERα36-stimulated ERK1/2 activation and/or ERα36 degradation in breast cancer cells, likely because its unique 27-amino acid domain replaces the last 5 (8–12) out of the 12 helixes in the ligand-binding domain of ERα66. Surprisingly, the authors reported also that exposure of cells to either the antiestrogen induced an ERα36-mediated activation of ERK1/2 even to a greater extent than that obtained with estradiol alone.

## 6. Estrogen Signaling in HCC

Multiple evidence from our own studies and other reports indicate that locally elevated expression and/or activity of the aromatase enzyme and the resulting high estrogen formation eventually lead to increasing AREG levels in human liver through an ER-mediated mechanism. This may in turn promote liver cancer cell growth through activation of EGFR and the ensuing MAPK/ERK signaling. Interestingly enough, according to what we have observed, AREG has been found as undetectable in normal liver, while but it could be promptly expressed in consequence of acute liver injury, behaving as a powerful regenerative and survival factor [81]. There is consistent evidence that AREG expression is raised in an array of chronic inflammatory diseases and various human cancers, including HCC [82]. In this context, Berasain et al. [81] have proposed that AREG plays a unique *nonredundant* role in the maintenance of the neoplastic phenotype of liver tumor cells and in HCC development and progression. Previous studies have reported that AREG expression is upregulated by estradiol in human breast cancer cells [83] and it is transcriptionally induced by estrogen in the mammary glands of pubertal mice at a time of exponential expansion of the ductal system [84]. Notably, La Marca and Rosen [85] have emphasized the potential role of AREG as a critical estrogen-induced paracrine regulator not only of mammary duct elongation, but also of mammary stem cell self-renewal and differentiation, suggesting that a switch from an ER-mediated, AREG paracrine activity to an autocrine pathway could represent a critical step of mammary carcinogenesis and tumor progression.

In our studies, we have suggested that locally elevated, aromatase-driven estrogen formation in human liver cancer tissues and cells may promote tumor cell growth through an estrogen-induced, ERα-mediated rise of AREG expression and the resulting activation of the EGFR signaling. In particular, we have observed that both AREG and its major ectodomain sheddase ADAM17 are correspondingly expressed with aromatase and ERα36 in human liver tissues and liver cancer cells.

There is accumulating evidence that neurofibromin II or Merlin, the product of the neurofibromatosis type 2 (NF2) gene, act as a “wizard” regulator of liver stem/progenitor cell proliferation [86], also through EGFR internalization and the activation of EGFR signaling pathways, which are responsible for the maintenance of tissue homeostasis. In an elegant study, Benhamouche and colleagues [66] established a conditional NF2/KO mice carrying liver-specific deletion of the NF2 tumor suppressor gene, demonstrating that a dramatic expansion of stem/progenitor cells occurs in either developing or adult mouse liver, with no impact on terminally differentiated hepatocytes. All mice outliving 30 weeks of age eventually develop either cholangiocellular or hepatocellular carcinoma, suggesting that NF2−/− liver stem/progenitors cells could represent tumor-initiating cells in both neoplasms. Based on this evidence, the authors speculate that NF2/Merlin may act as a critical regulator of cell contacts and growth signaling in the liver stem/progenitor cell niche. We have recently investigated Merlin expression in nontumoral, cirrhotic and malignant human liver tissues, showing that the product of NF2 tumor suppressor gene is expressed consistently with aromatase, ERα36, AREG and ADAM17 and that it is induced by estrogen in liver cancer cells [54].

**Figure 7:**
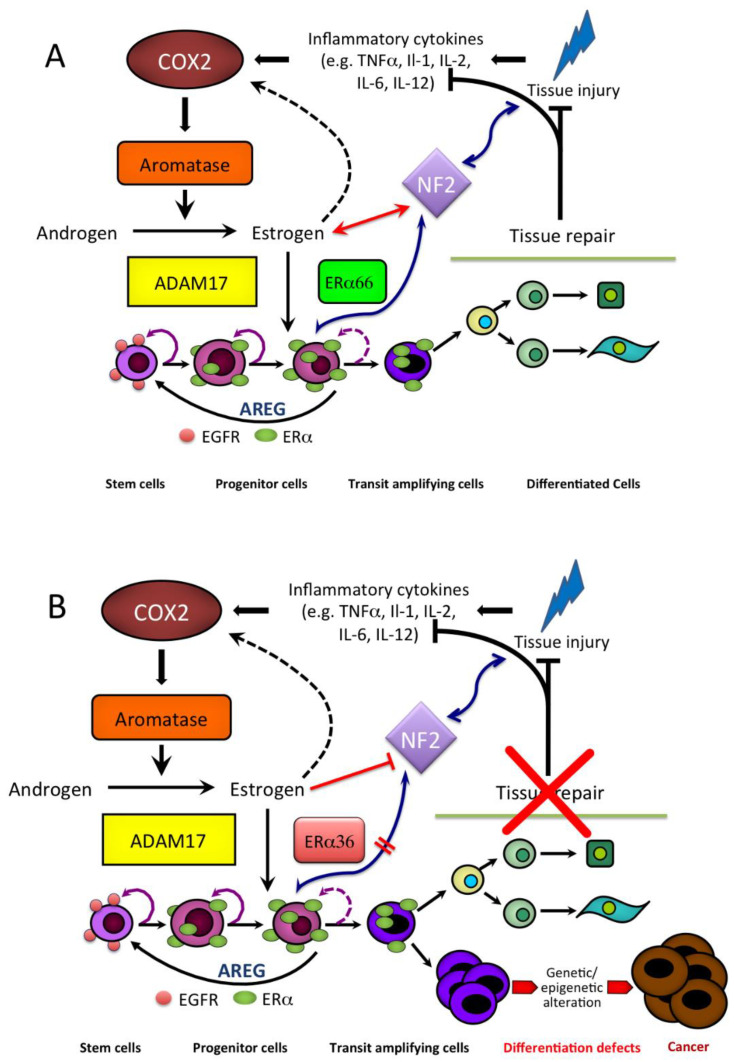
Hypothetical model for implication of aromatase-driven estrogen formation in both liver tissue injury and repair and cancer development: (**A**) In physiological condition, the proposed mechanism is activated in response to liver tissue injury through aromatase-driven estrogen formation, ultimately leading to growth/differentiation of stem/progenitor cells, repair of tissue damage, preservation of tissue homeostasis, and the return of the whole machinery to the initial state; (**B**) However, whether the occurrence of genetic/epigenetic alteration results in an impairment of stem/progenitor cell terminal differentiation, tissue damage cannot be repaired, the machinery remains persistently activated, potentially leading to the onset of either benign or malignant liver lesions in consequence of additional genetic/epigenetic changes reproduced with permission from [54].

Based on this combined evidence, we have proposed an estrogen-regulated mechanism of liver tissue injury and repair (see Figure 7) whereby, under physiological conditions, liver damage, either physical, chemical or biological, results in the establishment of an inflammatory microenvironment and a concurrent rise of aromatase expression and activity, leading to locally elevated estrogen formation. This latter produces an estrogen-induced, ERα66-mediated increase of AREG that could in turn be responsible for clonal expansion and terminal differentiation of stem/progenitor cells, repair of tissue damage, culminating with termination of the whole machinery and the outcome of tissue homeostasis preservation. In this framework, Merlin may represent a gatekeeper protein, sensing both tissue damage and aromatase-driven estrogen formation and acting in association with AREG in the regulation of stem/progenitor cell expansion and differentiation. However, the occurrence of an epigenetic alteration may result in a switch from the wild-type ERα66 to the splice variant ERα36 and a disruption of the terminal differentiation, eventually leading to the persistence of abnormally high aromatase, elevated estrogen, and an ERα36-mediated nongenomic signaling. The inability of liver stem/progenitor cells to terminally differentiate eventually leads to the abnormal expansion of liver stem cell niche, failure to repair tissue damage and, hence, a continued activation of the whole mechanism. The aberrant and sustained amplification of stem cell progeny may represent a basis for developing either benign or malignant liver cell growth as a result of accumulation of further genetic/epigenetic alteration. In this context, both local estrogen formation and Merlin expression remain persistently elevated in consequence of the disturbed liver stem cell niche and unrepaired tissue damage.

## 7. Estrogen and the Forkhead bOX Protein A in HCC

There is indication that sexual dimorphism in human liver is strictly dependent on the evolutionarily conserved Forkhead bOX protein A (FOXA), FOXA1 and FOXA2 [87,88]. These are also known as pioneer transcription factors in liver specification, with their DNA binding being described as an early event in the transcriptional regulation of several, context-specific, developmental processes of hormone-dependent organs and tissues, including liver [89]. Li and associates [90] reported that sexual dimorphism of liver cancer is completely abrogated in FOXA1- and FOXA2-deficient mice carrying chemically induced HCC.

**Figure 8:**
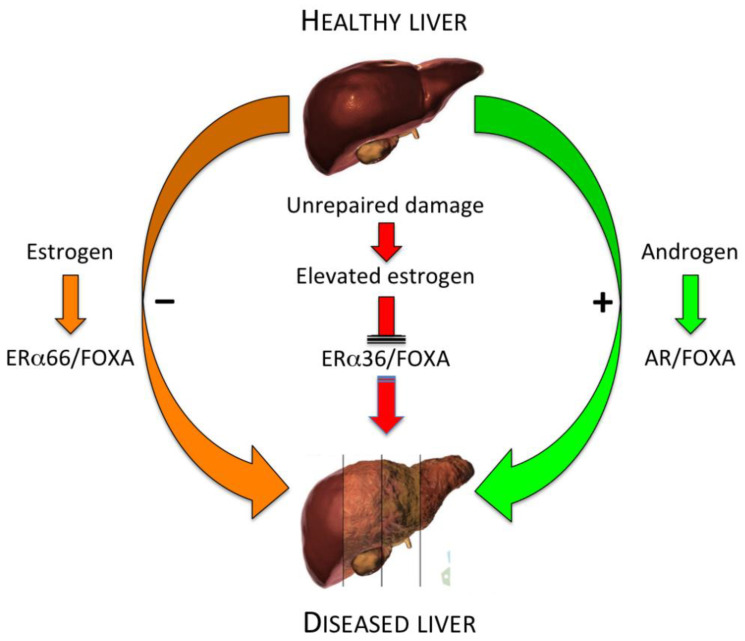
Speculative model of FOXA interaction with estrogen and androgen receptor in healthy and diseased liver. This schematic representation illustrates the potential interaction of FOXA transcription factors with both wild-type estrogen (ERα66) and androgen (AR) receptors, respectively in the prevention or promotion of liver diseases, including HCC. The hypothetical role of the ERα36 splice variant in the abrogation of ER/FOXA-mediated estrogen protective effects is also highlighted (modified and adapted from [88]).

Genome-wide studies have revealed that FOXA1 and ERα or androgen receptor (AR) frequently bind to adjacent cis-regulatory elements of respective target genes in human breast or prostate cancer cells and that the direct association of ER to chromatin occurs solely in the presence of Forkhead factor binding in close proximity [91]. In addition, the authors observed that specifically targeted knockdown of FOXA1 results in the abrogation of both ER chromatin binding and estrogen-induced gene expression, indicating that FOXA1 is required for the accomplishment of estrogen action in cancer cells. In this context, is it intriguing to speculate that the emergence of the ERα36 splicing variant may impair FOXA/ERα dual regulation of estrogen target genes and, consequently, be implicated in liver carcinogenesis and/or tumor progression (see Figure 8).

Major evidence reported in both this section and in Section 3, Section 4, Section 5 and Section 6 are summarized in Table 2.

## 8. Conclusions and Perspectives

Estrogens compose a superfamily of pleiotropic agents that act as key players in development, function, and homeostatic control of an amazing array of tissues, organs, and systems, to a point that estrogens can be regarded, in many respects, as “all season” hormones.

Tissue aromatase represents the rate-limiting enzyme responsible for the irreversible reaction converting the C19 androgenic steroid (testosterone and androstenedione) to the corresponding estrogen (respectively, estradiol and estrone). Aromatase is an important member of the P450 cytochrome superfamily of steroid enzymes that presides over estrogen formation in a variety of classical and non-classical target tissues, including liver. Aromatase-driven local estrogen production regulates a number of physiological processes, including target gene expression, protein biosynthesis, cell proliferation and differentiation, intercellular adhesion and communication, and so forth. Since aromatase is intrinsically implicated in a large collection of signaling pathways governing cell growth and differentiation at various target tissue and organs, it is likely that altered aromatase expression and/or activity may have a role in various human diseases [92]. In particular, there is sparse but consistent evidence that estrogens have important protective effects against the development of an assortment of chronic ailments, including neuro-degenerative and -inflammatory diseases (trauma, Alzheimer, Parkinson, and multiple sclerosis) [93,94], osteoarthritis and rheumatoid arthritis [95,96], polycystic ovarian syndrome [97], hemorrhage and thrombosis, endometriosis [98], obesity, diabetes, and hormone-related tumors (breast, prostate, ovary) [99].

**Figure 9:**
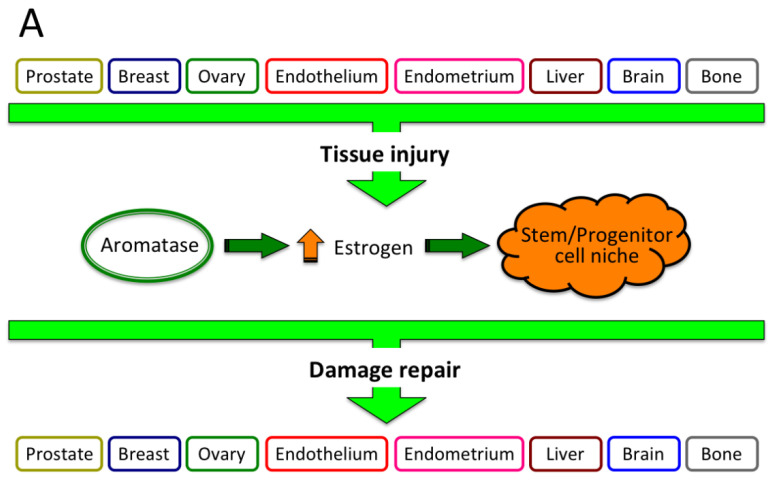

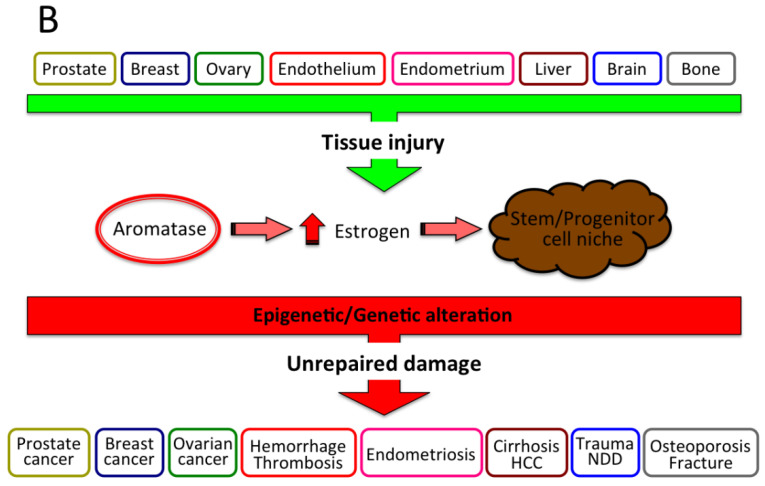
Unifying pathogenetic mechanism initiating various human chronic diseases. This diagram depicts a hypothetical pathogenetic mechanism featuring an array of chronic human diseases, whereby tissue insults, of any origin, eventually lead to an increase of aromatase activity, raise estrogen formation and address stem/progenitor cell niche for terminal differentiation and repair (*restitutio in integrum*) of tissue damage (**A**). However, whether the aromatase-estrogen-stem cell axis is disrupted, also because of genetic/epigenetic alteration, unrepaired, chronically persistent tissue damage may ultimately turn into either benign or malignant chronic disease(s), including hormone-related tumors (breast, prostate, ovary), polycystic ovary syndrome, hemorrhage and thrombosis, endometriosis, chronic liver diseases and HCC, neurodegenerative diseases (NDD: Alzheimer, schizophrenia, multiple sclerosis), osteoporosis and fracture (**B**).

In this context, the potential disruption of the aromatase/estrogen-dependent model of tissue injury and repair we have proposed, combined also with epigenetic/genetic alteration of cell growth/differentiation, may eventually lead to development and/or progression of various human diseases. Despite these diseases are of multifactorial origin, underlying mechanism(s) may, at least partly, recognize a common pathogenetic process featured by disruption of cell/tissue differentiation, impairment of tissue damage repair and continued, abnormally high aromatase/estrogen, as illustrated in Figure 9.

**Figure 10:**
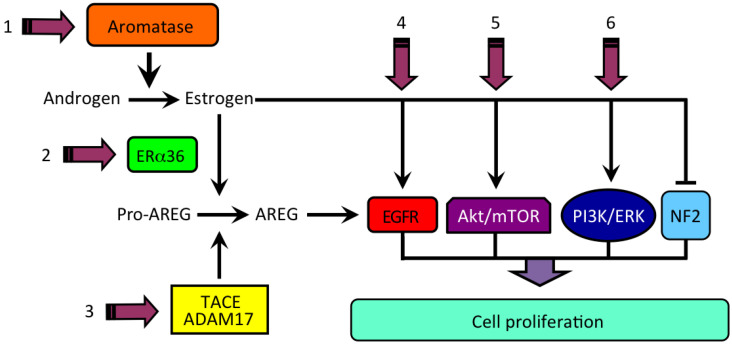
Hypothetical model for estrogen-regulated growth of HCC and potential sites of interference. We propose that elevated estrogen, produced by androgen aromatization, may be primarily implicated in human HCC through an ERα36-mediated induction of amphiregulin (AREG), binding to EGFR, and the activation of alternative signaling pathways, including Akt/mTOR, PI3K/ERK, or the disturbance of tumor suppressor gene product NF2 (neurofibromin 2/Merlin). (1) aromatase inhibitor (e.g., letrozole); (2) ER antagonist (e.g., ICI-182); (3) ADAM17 inhibitor (e.g., TAPI-2); (4) EGFR inhibitor (e.g., gefitinib); (5) Akt/mTOR inhibitor (e.g., MK2206/Everolimus); (6) PI3K/ERK inhibitor (e.g., XL147/SCH772984).

As far as human liver malignancy HCC is concerned, the multiple, though dispersed, evidence herein reviewed may represent an experimental basis for developing new therapeutic approaches and options to overcome either the unfavorable prognosis of advanced, inoperable HCC or the failure of hormonal systemic treatment (e.g., tamoxifen) or both. Although mechanism(s) underlying liver carcinogenesis and tumor progression remain largely unresolved, evidence resulting from studies on sex disparity in the occurrence and severity of various human liver diseases, including cancer, has provided a fertile ground to design and validate targeted therapeutic strategies for HCC patients. In this framework, the hypothetical model we have proposed for the implication of aromatase-driven elevated estrogen formation and persisting signaling in liver cancer development and/or progression may offer promising areas of translational and clinical research. Some issues potentially relevant for experimenting and trialing new treatment avenues for the management of HCC patients are illustrated in Figure 10.

In conclusion, the currently accumulated experimental evidence is highly suggestive for the protective role of estrogen in most chronic liver diseases, including cancer; on the other hand, however, disruption of the delicate balance and complex machinery relating estrogen, signaling mechanisms, stem/progenitor cells, and liver homeostasis may well be responsible for the formation and maintenance of a persisting inflammatory state, ultimately leading to the onset of chronic diseases, notably HCC.

## Figures and Tables

**Table 1:** Summary data on the protective role of estrogen in liver diseases.

Liver Disease	Risk	Mechanism(s)	Reference
Steatosis	↓	↓ lipogenesis and fatty acid uptake ↑ lipolysis and cholesterol secretion	Shen & Shi, 2015 [6]
Steatohepatitis	↑	TAM treatment of breast cancer patients	Yoo et al., 2020 [8]
Fatty liver	↑	Estrogen deficiency in ArKO mouse	Jones et al., 2000 [9]
Steatosis	↑	Deletion of hepatic ERα	Palmisano et al., 2016 [12]
Fatty liver	↓	↓ de novo hepatic lipogenesis	Gao et al., 2008 [13]
Steatosis	↓	↓ cholesterol biosynthesis and uptake	Palmisano et al., 2017 [15]
Steatosis	↓	↓ delivery of adipose FA to liver	Otero et al., 2014 [17]
Trauma/Injury	↓	↓ proinflammatory cytokines ↑ hepatic hemeoxygenase-1 ↑ HSP 32 and HSP 70	Yokohama et al., 2003 [19] Hsu et al., 2007 [20] Szalay et al., 2006 [21]
Fibrosis/NASH	↑	Postmenopausal estrogen deprivation	Villa et al., 2012 [23] Yang et al., 2014 [25]
NAFLD	↑	↑ duration of estrogen deficiency	Klair et al., 2016 [30]
NAFLD	↑	Nonsteroidal aromatase inhibitors treatment of breast cancer patients	Lee at al., 2019 [31]
HBV	↓	Estrogen antioxidant activity	Shimizu et al., 2007 [34]
HCV	↓	↓ production of mature HCV and HCV cell entry	Ruggieri et al., 2018 [35]
HCC	↓	Binding and activation of ERβ by estradiol	Iavarone et al., 2003 [37]

TAM, tamoxifen; ERα, estrogen receptor α; FA, fatty acids; NASH, nonalcoholic steatohepatitis; NAFLD, nonalcoholic fatty liver disease; HBV, chronic hepatitis B virus; HCV, chronic hepatitis C virus; HCC, hepatocellular carcinoma.

**Table 2:** Summary data on the potential implication of estrogen in chronic liver disease and hepatocellular carcinoma.

Liver Disease	Risk	Mechanism(s)	Reference
HBV	↑	↑ in situ estrogen synthesis	Murakami et al., 2020 [49]
HCC	↑	Hypermethylation of the ESR1 gene	Hishida et al., 2013 [46]
HCC	↑	Variant estrogen receptor(s)	Villa et al., 1995 [47]
Cirrhosis/HCC	↑	ERα36 splice variant	Miceli et al., 2011 [55]
HCC	↑	Promoter-driven ↑ aromatase expression	Koh et al., 2011 [48]
HCC	↑	↑ aromatase expression and activity	Catagnetta et al., 2003 [50]
HCC	↑	Estrogen-induced ↑ AREG expression	Carruba et al., 2011 [52]
HCC	↑	AREG-induced ↑ liver cancer cell growth	Cocciadiferro et al., 2017 [54]
HCC	↑	Liver-specific deletion of NF2 (NF2/KO)	Benhamouche et al., 2010 [66]
HCC	↓	NF2 (Merlin) regulation of liver stem/progenitor cell niche	Villanueva, 2010 [86]
HCC	↑	Persistently ↑ estrogen formation and NF2 (Merlin) upregulation	Cocciadiferro et al., 2017 [54]
HCC	↓	FOXA-dependent ERα-mediated estrogen signaling	Zhao & Li, 2015 [88]
HCC	↑	FOXA1/2-deficient mice	Li et al., 2012 [90]

HBV, chronic hepatitis B virus; HCV, chronic hepatitis C virus; HCC, hepatocellular carcinoma; ESR1, estrogen steroid receptor 1; ERα36, estrogen receptor α 36; AREG, amphiregulin; NF2, neurofibromatosis type 2; FOXA, Forkhead bOX protein A.

## Data Availability

No new data were created or analyzed in this study. Data sharing is not applicable to this article.

## References

[1] Mauvais-Jarvis F., Clegg D.J., Hevener A.L. (2013). The Role of Estrogens in Control of Energy Balance and Glucose Homeostasis. Endocr. Rev..

[2] Laroche G. (1953). Liver and sex hormones. Rev. Int. Hepatol..

[3] Barzilai D. (1965). The effect of the sex hormones on liver physiology and pathology. Acta Hepato-Splenol..

[4] Maggi A., Della Torre S. (2018). Sex, metabolism and health. Mol. Metab..

[5] Kur P., Kolasa-Wołosiuk A., Misiakiewicz-Has K., Wiszniewska B. (2020). Sex Hormone-Dependent Physiology and Diseases of Liver. Int. J. Environ. Res. Public Health.

[6] Shen M., Shi H. (2015). Sex Hormones and Their Receptors Regulate Liver Energy Homeostasis. Int. J. Endocrinol..

[7] Parthasarathy C., Renuka V.N., Balasubramanian K. (2009). Sex steroids enhance insulin receptors and glucose oxidation in Chang liver cells. Clin. Chim. Acta.

[8] Yoo J.-J., Lim Y.S., Kim M.S., Lee B., Kim B.-Y., Kim Z., Lee J.E., Lee M.H., Kim S.G., Kim Y.S. (2020). Risk of fatty liver after long-term use of tamoxifen in patients with breast cancer. PLoS ONE.

[9] Jones M.E., Thorburn A.W., Britt K.L., Hewitt K.N., Wreford N.G., Proietto J., Oz O.K., Leury B.J., Robertson K.M., Yao S. (2000). Aromatase-deficient (ArKO) mice have a phenotype of increased adiposity. Proc. Natl. Acad. Sci. USA.

[10] Hewitt K.N., Pratis K., Jones M.E.E., Simpson E.R. (2004). Estrogen Replacement Reverses the Hepatic Steatosis Phenotype in the Male Aromatase Knockout Mouse. Endocrinology.

[11] Chow J.D., Jones M.E., Prelle K., Simpson E.R., Boon W.C. (2011). A selective estrogen receptor alpha agonist ameliorates hepatic steatosis in the male aromatase knockout mouse. J. Endocrinol..

[12] Palmisano B.T., Le T.D., Zhu L., Lee Y.K., Stafford J.M. (2016). Cholesteryl ester transfer protein alters liver and plasma triglyceride metabolism through two liver networks in female mice. J. Lipid Res..

[13] Gao H., Falt S., Sandelin A., Gustafsson J.A., Dahlman-Wright K. (2008). Genome-wide identification of estrogen receptor alpha-binding sites in mouse liver. Mol. Endocrinol..

[14] Zhang H., Liu Y., Wang L., Li Z., Zhang H., Wu J., Rahman N., Guo Y., Li D., Li N. (2013). Differential effects of estrogen/androgen on the prevention of nonalcoholic fatty liver disease in the male rat. J. Lipid Res..

[15] Palmisano B.T., Zhu L., Stafford J.M. (2017). Role of Estrogens in the Regulation of Liver Lipid Metabolism. Adv. Exp. Med. Biol..

[16] Barros R.P., Gustafsson J.-Å. (2011). Estrogen Receptors and the Metabolic Network. Cell Metab..

[17] Otero Y.F., Stafford J.M., McGuinness O.P. (2014). Pathway-selective Insulin Resistance and Metabolic Disease: The Importance of Nutrient Flux. J. Biol. Chem..

[18] Bösch F., Angele M.K., Chaudry I.H. (2018). Gender differences in trauma, shock and sepsis. Mil. Med Res..

[19] Yokoyama Y., Kuebler J.F., Matsutani T., Schwacha M.G., Bland K.I., Chaudry I.H. (2003). Mechanism of the salutary effects of 17beta-estradiol following trauma-hemorrhage: Direct downregulation of Kupffer cell proinflammatory cytokine production. Cytokine.

[20] Hsu J.-T., Kan W.-H., Hsieh C.-H., Choudhry M.A., Schwacha M.G., Bland K.I., Chaudry I.H. (2007). Mechanism of estrogen-mediated attenuation of hepatic injury following trauma-hemorrhage: Akt-dependent HO-1 up-regulation. J. Leukoc. Biol..

[21] Szalay L., Shimizu T., Suzuki T., Yu H.P., Choudhry M.A., Schwacha M.G., Rue L.W., Bland K.I., Chaudry I.H. (2006). Estradiol improves cardiac and hepatic function after trauma-hemorrhage: Role of enhanced heat shock protein expression. Am. J. Physiol. Regul. Integr. Comp. Physiol..

[22] Charni-Natan M., Aloni-Grinstein R., Osher E., Rotter V. (2019). Liver and Steroid Hormones-Can a Touch of p53 Make a Difference?. Front. Endocrinol..

[23] Villa E., Vukotic R., Cammà C., Petta S., Di Leo A., Gitto S., Turola E., Karampatou A., Luisa Losi Bernabucci V., Cenci A. (2012). Reproductive status is associated with the severity of fibrosis in women with hepatitis C. PLoS ONE.

[24] Cengiz M., Ozenirler S., Yılmaz G. (2014). Estrogen Receptor Alpha Expression and Liver Fibrosis in Chronic Hepatitis C Virus Genotype 1b: A Clinicopathological Study. Zahedan J. Res. Med Sci..

[25] Yang J.D., Abdelmalek M.F., Pang H., Guy C.D., Smith A.D., Diehl A.M., Suzuki A. (2014). Gender and Menopause Impact Severity of Fibrosis Among Patients with Nonalcoholic Steatohepatitis. Hepatology.

[26] Yasuda M., Shimizu I., Shiba M., Ito S. (1999). Suppressive effects of estradiol on dimethylnitrosamine-induced fibrosis of the liver in rats. Hepatology.

[27] Itagaki T., Shimizu I., Cheng X., Yuan Y., Oshio A., Tamaki K., Fukuno H., Honda H., Okamura Y., Ito S. (2005). Opposing effects of oestradiol and progesterone on intracellular pathways and activation processes in the oxidative stress induced activation of cultured rat hepatic stellate cells. Gut.

[28] Lee C., Kim J., Jung Y. (2019). Potential therapeutic application of estrogen in gender disparity of nonalcoholic fatty liver disease/nonalcoholic steatohepatitis. Cells.

[29] Lonardo A., Nascimbeni F., Ballestri S., Fairweather D., Win S., Than T.A., Abdelmalek M.F., Suzuki A. (2019). Sex Differences in Nonalcoholic Fatty Liver Disease: State of the Art and Identification of Research Gaps. Hepatology.

[30] Klair J.S., Yang J.D., Abdelmalek M.F., Guy C., Gill R., Yates K., Unalp-Arida A., Lavine J., Clark J., Diehl A.M. (2016). A longer duration of estrogen deficiency increases fibrosis risk among postmenopausal women with nonalcoholic fatty liver disease. Hepatology.

[31] Lee J.I., Yu J.H., Anh S.G., Lee H.W., Jeong J., Lee K.S. (2019). Aromatase inhibitors and newly developed nonalcoholic fatty liver disease in postmenopausal patients with early breast cancer: A propensity score-matched cohort study. Oncologist.

[32] Della Torre S. (2020). Non-alcoholic Fatty Liver Disease as a Canonical Example of Metabolic Inflammatory-Based Liver Disease Showing a Sex-Specific Prevalence: Relevance of Estrogen Signaling. Front. Endocrinol..

[33] Poyard T., Ratziu V., Charlotte F., Goodman Z., McHutchinson J., Albrecht J. (2001). Rates and risk factors of liver fibrosis progression in patients with chronic hepatitis C. J. Hepatol..

[34] Shimizu I., Kohno N., Tamaki K., Shono M., Huang H.W., He J.H., Yao D.F. (2007). Female hepatology: Favorable role of estrogen in chronic liver disease with hepatitis B virus infection. World J. Gastroenterol..

[35] Ruggieri A., Gagliardi M.C., Anticoli S. (2018). Sex-Dependent Outcome of Hepatitis B and C Viruses Infections: Synergy of Sex Hormones and Immune Responses?. Front. Immunol..

[36] Brady C.W. (2015). Liver disease in menopause. World J. Gastroenterol..

[37] Iavarone M., Lampertico P., Seletti C., Donato M.F., Ronchi G., del Ninno E., Colombo M. (2003). The clinical and pathogenetic significance of estrogen receptor beta expression in chronic liver diseases and liver carcinoma. Cancer.

[38] ACS American Cancer Society (2018). Global Cancer: Facts and Figures.

[39] AIOM (2020). I Numeri del Cancro in Italia 2020.

[40] Nagasue N., Kohno H. (1992). Hepatocellular Carcinoma and Sex Hormones. HPB Surg..

[41] Kalra M., Mayes J., Assefa S., Kaul A.K., Kaul R. (2008). Role of sex steroid receptors in pathobiology of hepatocellular carcinoma. World J. Gastroenterol..

[42] Ohnishi S., Murakami T., Moriyama T., Mitamura K., Imawari M. (1986). Androgen and estrogen receptors in hepatocellular carcinoma and in the surrounding noncancerous liver tissue. Hepatology.

[43] Engstrom P.F., Levin B., Moertel C.G., Schutt A. (1990). A phase II trial of tamoxifen in hepatocellular carcinoma. Cancer.

[44] CLIP-Group (1998). Tamoxifen in treatment of hepatocellular carcinoma: A randomized controlled trial. CLIP-Group (Cancer of the Liver Italian Programme). Lancet.

[45] Di Maio M., Daniele B., Pignata S., Gallo C., De Maio E., Morabito A., Piccirillo M.-C., Perrone F. (2008). Is human hepatocellular carcinoma a hormone-responsive tumor?. World J. Gastroenterol..

[46] Hishida M., Nomoto S., Inokawa Y., Hayashi M., Kanda M., Okamura Y., Nishikawa Y., Tanaka C., Kobayashi D., Yamada S. (2013). Estrogen receptor 1 gene as a tumor suppressor gene in hepatocellular carcinoma detected by triple-combination array analysis. Int. J. Oncol..

[47] Villa E., Camellini L., Dugani A., Zucchi F., Grottola A., Merighi A., Buttafoco P., Losi L., Manenti F. (1995). Variant estrogen receptor messanger RNA species detected in human primary hepatocellular carcinoma. Cancer Res..

[48] Koh W.-P., Yuan J.-M., Wang R., Govindarajan S., Oppenheimer R., Zhang Z.Q., Yu M.C., Ingles S.A. (2011). Aromatase (CYP19) promoter gene polymorphism and risk of nonviral hepatitis-related hepatocellular carcinoma. Cancer.

[49] Murakami K., Hata S., Miki Y., Sasano H. (2018). Aromatase in normal and diseased liver. Horm. Mol. Biol. Clin. Investig..

[50] Castagnetta L.A.M., Agostara B., Montalto G., Polito L., Campisi I., Saetta A., Itoh T., Yu B., Chen S., Carruba G. (2003). Local estrogen formation by nontumoral, cirrhotic, and malignant human liver tissues and cells. Cancer Res..

[51] Carruba G. (2009). Aromatase in Nontumoral and Malignant Human Liver Tissues and Cells. Ann. N. Y. Acad. Sci..

[52] Carruba G., Miceli V., Cocciadiferro L., Zarcone M., Agostara B., Montalto G., Granata O.M. (2011). Estrogen signalling through amphiregulin may be implicated in human hepatocellular carcinoma. Horm. Mol. Biol. Clin. Investig..

[53] Hosur V., Farley M.L., Burzenski L.M., Shultz L.D., Wiles M.V. (2018). ADAM17 is essential for ectodomain shedding of the EGF-receptor ligand amphiregulin. FEBS Open Bio.

[54] Cocciadiferro L., Miceli V., Granata O.M., Carruba G. (2017). Merlin, the product of NF2 gene, is associated with aromatase expression and estrogen formation in human liver tissues and liver cancer cells. J. Steroid. Biochem. Mol. Biol..

[55] Miceli V., Cocciadiferro L., Fregapane M., Zarcone M., Montalto G., Polito L.M., Agostara B., Granata O.M., Carruba G. (2011). Expression of wild-type and variant estrogen receptor alpha in liver carcinogenesis and tumor progression. Omics J. Integr. Biol..

[56] McClatchey A.I., Fehon R.G. (2009). Merlin and the ERM proteins—Regulators of receptor distribution and signaling at the cell cortex. Trends Cell Biol..

[57] Chiasson-MacKenzie C., Morris Z.S., Baca Q., Morris B., Coker J.K., Mirchev R., Jensen A.E., Carey T., Stott S.L., Golan D.E. (2015). NF2/Merlin mediates contact-dependent inhibition of EGFR mobility and internalization via cortical actomyosin. J. Cell Biol..

[58] Harvey K.F., Zhang X., Thomas D.M. (2013). The Hippo pathway and human cancer. Nat. Rev. Cancer.

[59] Barron D., Kagey J.D. (2014). The role of the Hippo pathway in human disease and tumorigenesis. Clin. Transl. Med..

[60] Hamaratoglu F., Willecke M., Kango-Singh M., Nolo R., Hyun E., Tao C., Jafar-Nejad H., Halder G. (2005). The tumour-suppressor genes NF2/Merlin and Expanded act through Hippo signalling to regulate cell proliferation and apoptosis. Nat. Cell Biol..

[61] Morrow K.A., Shevde L.A. (2012). Merlin: The wizard requires protein stability to function as a tumor suppressor. Biochim. Biophys. Acta.

[62] Shaw R.J., Paez J.G., Curto M., Yaktine A., Pruitt W.M., Saotome I., O’Bryan J.P., Gupta V., Ratner N., Der C.J. (2001). The Nf2 tumor suppressor, merlin, functions in Rac-dependent signaling. Dev. Cell.

[63] Kissil J.L., Wilker E.W., Johnson K.C., Eckman M.S., Yaffe M.B., Jacks T. (2003). Merlin, the Product of the Nf2 Tumor Suppressor Gene, Is an Inhibitor of the p21-Activated Kinase, Pak1. Mol. Cell.

[64] Curto M., Cole B.K., Lallemand D., Liu C.-H., McClatchey A.I. (2007). Contact-dependent inhibition of EGFR signaling by Nf2/Merlin. J. Cell Biol..

[65] James M.F., Han S., Polizzano C., Plotkin S.R., Manning B.D., Stemmer-Rachmanimov A.O., Gusella J.F., Ramesh V. (2009). NF2/merlin is a novel negative regulator of mTOR complex 1, and activation of mTORC1 is associated with meningioma and schwannoma growth. Mol. Cell Biol..

[66] Benhamouche S., Curto M., Saotome I., Gladden A.B., Liu C.-H., Giovannini M., McClatchey A.I. (2010). Nf2/Merlin controls progenitor homeostasis and tumorigenesis in the liver. Genes Dev..

[67] Duffy M. (1978). Estradiol receptors in human liver. J. Steroid Biochem..

[68] Nagasue N., Yukaya H., Ogawa Y., Ito A. (1986). Estrogen receptors in hepatocellular carcinoma. Cancer.

[69] Farinati F., Salvagnini M., De Maria N., Fornasiero A., Chiaramonte M., Rossaro L., Naccarato R. (1990). Unresectable hepatocellular carcinoma: A prospective controlled trial with tamoxifen. J. Hepatol..

[70] Boix L., Bruix J., Castells A., Fuster J., Bru C., Visa J., Rivera F., Rodes J. (1993). Sex hormone receptors in hepatocellular carcinoma. Is there a rationale for hormonal treatment?. J. Hepatol..

[71] Martínez Cerezo F.J., Tomás A., Donoso L., Enríquez J., Guarner C., Balanzó J., Nogueras A.M., Vilardell F. (1994). Controlled trial of tamoxifen in patients with advanced hepatocellular carcinoma. J. Hepatol..

[72] Simonetti R.G., Liberati A., Angiolini C., Pagliaro L. (1997). Treatment of hepatocellular carcinoma: A systematic review of randomized controlled trials. Ann. Oncol..

[73] Nowak A.K., Stockler M.R., Chow P.K.H., Findlay M. (2005). Use of tamoxifen in advanced-stage hepatocellular carcinoma. Cancer.

[74] Gallo C., De Maio E., Di Maio M., Signoriello G., Daniele B., Pignata S., Annunziata A., Perrone F. (2006). Tamoxifen is not effective in good prognosis patients with hepatocellular carcinoma. BMC Cancer.

[75] Salhab M., Canelo R. (2011). An overview of evidence-based management of hepatocellular carcinoma: A meta-analysis. J. Cancer Res. Ther..

[76] Wang Z., Zhang X., Shen P., Loggie B.W., Chang Y., Deuel T.F. (2005). Identification, cloning, and expression of human estrogen receptor-α36, a novel variant of human estrogen receptor-α66. Biochem. Biophys. Res. Commun..

[77] Wang Z., Zhang X., Shen P., Loggie B.W., Chang Y., Deuel T.F. (2006). A variant of estrogen receptor-α, hERα36: Transduction of estrogen- and antiestrogen-dependent membrane-initiated mitogenic signaling. Proc. Natl. Acad. Sci. USA.

[78] Shi L., Dong B., Li Z., Lu Y., Ouyang T., Li J., Wang T., Fan Z., Fan T., Lin B. (2009). Expression of ER-α36, a Novel Variant of Estrogen Receptor α, and Resistance to Tamoxifen Treatment in Breast Cancer. J. Clin. Oncol..

[79] Pagano M.T., Ortona E., Dupuis M.L. (2020). A Role for Estrogen Receptor alpha36 in Cancer Progression. Front. Endocrinol..

[80] Li G., Zhang J., Jin K., He K., Zheng Y., Xu X., Wang H., Wang H., Li Z., Yu X. (2013). Estrogen receptor-α36 is involved in development of acquired tamoxifen resistance via regulating the growth status switch in breast cancer cells. Mol. Oncol..

[81] Berasain C., Castillo J., Perugorria M., Prieto J., Avila M. (2007). Amphiregulin: A new growth factor in hepatocarcinogenesis. Cancer Lett..

[82] Berasain C., Avila M.A. (2014). Amphiregulin. Semin. Cell Dev. Biol..

[83] Vendrell J.A., Magnino F., Danis E., Duchesne M.J., Pinloche S., Pons M., Birnbaum D., Nguyen C., Theillet C., Cohen P.A. (2004). Estrogen regulation in human breast cancer cells of new downstream gene targets involved in estrogen metabolism, cell proliferation and cell transformation. J. Mol. Endocrinol..

[84] Ciarloni L., Mallepell S., Brisken C. (2007). Amphiregulin is an essential mediator of estrogen receptor alpha function in mammary gland development. Proc. Natl. Acad. Sci. USA.

[85] Lamarca H.L., Rosen J.M. (2007). Estrogen regulation of mammary gland development and breast cancer: Amphiregulin takes center stage. Breast Cancer Res..

[86] Villanueva T. (2010). Merlin, the liver wizard. Nat. Rev. Cancer.

[87] Le Lay J., Kaestner K.H. (2010). The Fox Genes in the Liver: From Organogenesis to Functional Integration. Physiol. Rev..

[88] Zhao Y., Li Z. (2015). Interplay of estrogen receptors and FOXA factors in the liver cancer. Mol. Cell. Endocrinol..

[89] Hannenhalli S., Kaestner K.H. (2009). The evolution of Fox genes and their role in development and disease. Nat. Rev. Genet..

[90] Li Z., Tuteja G., Schug J., Kaestner K.H. (2012). Foxa1 and Foxa2 Are Essential for Sexual Dimorphism in Liver Cancer. Cell.

[91] Carroll J.S., Liu X.S., Brodsky A.S., Li W., Meyer C.A., Szary A.J., Eeckhoute J., Shao W., Hestermann E.V., Geistlinger T.R. (2005). Chromosome-Wide Mapping of Estrogen Receptor Binding Reveals Long-Range Regulation Requiring the Forkhead Protein FoxA1. Cell.

[92] Patel S. (2017). Disruption of aromatase homeostasis as the cause of a multiplicity of ailments: A comprehensive review. J. Steroid Biochem. Mol. Biol..

[93] Saldanha C.J., Duncan K.A., Walters B.J. (2009). Neuroprotective actions of brain aromatase. Front. Neuroendocr..

[94] Duncan K.A., Saldanha C.J. (2020). Central aromatization: A dramatic and responsive defense against threat and trauma to the vertebrate brain. Front. Neuroendocr..

[95] Hernández J.L., Garcés C.M., Sumillera M., Fernández-Aldasoro E.V., García-Ibarbia C., Ortiz-Gómez J.A., Arozamena J., Alonso M.A., Riancho J.A. (2008). Aromatase expression in osteoarthritic and osteoporotic bone. Arthritis Rheum..

[96] Castagnetta L.A., Carruba G., Granata O.M., Stefano R., Miele M., Schmidt M., Cutolo M., Straub R.H. (2003). Increased estrogen formation and estrogen to androgen ratio in the synovial fluid of patients with rheumatoid arthritis. J. Rheumatol..

[97] Rocha A.L., Oliveira F.R., Azevedo R.C., Silva V.A., Peres T.M., Candido A.L., Gomes K.B., Reis F.M. (2019). Recent advances in the understanding and management of polycystic ovary syndrome. F1000Research.

[98] Mori T., Ito F., Koshiba A., Kataoka H., Takaoka O., Okimura H., Khan K.N., Kitawaki J. (2019). Local estrogen formation and its regulation in endometriosis. Reprod. Med. Biol..

[99] Williams G.P. (2010). The role of oestrogen in the pathogenesis of obesity, type 2 diabetes, breast cancer and prostate disease. Eur. J. Cancer Prev..

